# phase1RMD: An R package for repeated measures dose-finding designs with novel toxicity and efficacy endpoints

**DOI:** 10.1371/journal.pone.0256391

**Published:** 2021-09-02

**Authors:** Jun Yin, Yu Du, Rui Qin, Shihao Shen, Sumithra Mandrekar

**Affiliations:** 1 Department of Health Sciences Research, Mayo Clinic, Rochester, MN, United States of America; 2 Statistics-Diabetes/Endocrine, Eli Lilly and Company, Indianapolis, IN, United States of America; 3 Clinical Biostatistics, Janssen Research and Development, Raritan, NJ, United States of America; 4 Data Science & Biostatistics, DermBiont, Boston, MA, United States of America; Goethe University Hospital Frankfurt, GERMANY

## Abstract

Traditional dose-finding designs are substantially inefficient for targeted agents and cancer immunotherapies by failing to incorporate efficacy signals, mild and moderate adverse events, and late, cumulative toxicities. However, the lack of user-friendly software is a barrier to the practical use of the novel phase I designs, despite their demonstrated superiority of traditional 3+3 designs. To overcome these barriers, we present an R package, phase1RMD, which provides a comprehensive implementation of novel designs with repeated toxicity measures and early efficacy. A novel phase I repeated measures design that used a continuous toxicity score from multiple treatment cycles was implemented. Furthermore, in studies where preliminary efficacy is evaluated, an adaptive, multi-stage design to identify the most efficacious dose with acceptable toxicity was demonstrated. Functions are provided to recommend the next dose based on the data collected in a phase I trial, as well as to assess trial characteristics given design parameters via simulations. The repeated measure designs accurately estimated both the magnitude and direction of toxicity trends in late treatment cycles, and allocated more patients at therapeutic doses. The R package for implementing these designs is available from the Comprehensive R Archive Network. To our best knowledge, this is the first software that implement novel phase I dose-finding designs that simultaneously accounts for the multiple-grade toxicity events over multiple treatment cycles and a continuous early efficacy outcome. With the software published on CRAN, we will pursue the implementation of these designs in phase I trials in real-life settings.

## 1. Introduction

Phase I clinical trials in oncology are designed to identify the recommended phase II dose (RP2D) for follow-up trials. The unique features of the Molecularly Targeted Agents (MTAs) and cancer immunotherapies require that novel toxicity endpoints are considered in the phase I oncology clinical trials. The toxicity profile of MTAs and cancer immunotherapies are often substantially different from cytotoxic agents, characterized by chronic, prolonged events or cumulative toxicity as opposed to the early onset adverse events associated with cytotoxic therapies. The prolonged treatment of MTAs and cancer immunotherapies deem it necessary to consider moderate toxicities with repeated occurrence in multiple treatment cycles.

The total toxicity profile (TTP) was developed as a new toxicity endpoint to address the over-simplification of toxicity data in phase I trials [1]. It is a compound toxicity score which absorbs multiple toxicities from one patient, by weighting toxicities by grade and type.

We further extended their approach to combine the multiple assessment obtained on a patient while the patient continues to receive treatment beyond the first cycle [2]: the repeated measures design (RMD). The RMD is an adaptive dose-finding design based on the longitudinal TTP scores. Compared to traditional 3+3 and recent CRM designs, the RMD models adverse event data from all cycles of treatment leading to a more robust estimation and dose selection. It also allows for patient drop-out once they experience a dose limiting toxicity (DLT) event or disease progression which are typically seen in a phase I study. The RMD design with multiple-cycle toxicity is implemented in this R package **phase1RMD**.

Traditionally, for conventional cytotoxic agents, the highest dose patients could tolerate was thought to produce the greatest clinical benefit. However, the recent increase in the knowledge of molecular mechanisms shows that molecular targeted agents (MTAs) might achieve clinical benefit at lower dose levels than those required with conventional cytotoxic agents [3, 4]. As a result, searching for the maximum-tolerated dose may no longer be the optimal treatment strategy for phase I oncology trials. It has become more important to consider early-stage efficacy endpoints in addition to a comprehensive toxicity profile for these novel agents.

To incorporate early efficacy signals, we further developed a three-stage dose-finding design based on both longitudinal toxicity data of TTP scores and an efficacy outcome. Stage 1 of the design uses toxicity data to perform dose-escalation and to identify a set of initially allowable (safe) doses; stage 2 of the design incorporates an efficacy outcome to update the set of allowable doses and randomizes the new cohort of patients to the allowable doses with emphasis towards those dose levels with a higher predicted efficacy; stage 3 uses all data from all treated patients at the end of the trial to make final recommendations. To our best knowledge, this is the first design that simultaneously accounts for the multiple-grade toxicity events over multiple treatment cycles and a continuous early efficacy outcome. The correlation between multiple-cycle toxicity TTP scores and efficacy outcomes are accounted for in a joint model with a compound symmetric covariance structure. The repeated measure model (RMD) with a dual-endpoint (multiple-cycle toxicity and early efficacy) is implemented in this R package.

Both the repeated measure design (RMD) on toxicity only and the RMD on the dual-endpoint are implemented in the Bayesian framework. Where historical data or elicitation are available, they can be used to describe prior beliefs about the shape and the uncertainty in the dose toxicity curve; and when no such data is available, the model uses non-informative priors. Escalation decisions are based on the posterior distribution of toxicity.

To implement the proposed designs, close collaboration between investigators and statisticians is required at the design stage. In addition, although the model-based designs have been demonstrated to be superior to the traditional 3+3 design, model-based designs have been perceived as black-box and many oncologists are reluctant to use the model-based designs as a result. The lack of user-friendly software is another barrier to the practical use of the model-based designs.

To overcome the above barriers, we present a R package **phase1RMD**, which provides a comprehensive implementation of the RMD design with toxicity only and the RMD design with dual endpoints. The package is available from the Comprehensive R Archive Network at https://cran.r-project.org/web/packages/phase1RMD/index.html. To our best knowledge, this is the first software that focuses explicitly on continuous toxicity endpoints such as the total toxicity profile (TTP). The main features of the **phase1RMD** are as follows:

1. The flexibility to change design inputs such as the cohort size, and algorithmic constraints placed on escalation.
2. The flexibility to change the prior distributions.
3. Easy to assess operating characteristics using built-in simulation code.
4. Two methods of simulating high dimensional adverse event data; both non-parametric approach and parametric approach in data generation are incorporated.
5. An interactive approach is possible with data entered and decisions made after each cohort is enrolled.
6. Informative text output from simulations or from conducting a single trial.
7. Estimation using the Markov chain Monte Carlo via WinBUGS/JAGS using the R pacakge **R2WinBUGS**/**rjags** [5, 6].

The aim of this article is to demonstrate the options available within the R package and to guide users through examples to interactively run a trial and evaluate trial designs via simulations. Section 1 describes and demonstrates the calculation of the novel toxicity endpoint, TTP, in the RMD designs. Section 2 demonstrates the RMD design with longitudinal TTP-based toxicity only while Section 3 illustrates the extension of RMD model to incorporate both longitudinal TTP-based toxicity and efficacy dual-endpoint. Section 4 concludes the article with remarks and recommendation for future research.

## 2. Methods

### 2.1 Quasi-continuous toxicity endpoint

The proposed RMD designs use a quasi-continuous toxicity score, the total toxicity profile (TTP), developed to overcome the over-simplification of toxicity data in phase I trials [1]. The TTP can capture multiple types and grades of toxicities occurring during the pre-specified interval. The *TTP*_*i*_ is defined as:

$$\begin{array}{c} TTP_{i}=\sqrt{\sum_{l=1}^{L}\sum_{h=0}^{4}w_{lh}^{2}1\left(G_{il}=h\right)}. \end{array}$$ (1)

Let *w*_*lh*_ denote the elicited weight of toxicity type *l* (*l* ∈ {1, …, *L*}) occurring at grade *h* (*h* ∈ {0, …, 4}). Hence, the weight vector for toxicity *l* is ***w*_*l*_** = (*w*_*l*0_, …, *w*_*l*4_)^⊤^ and the weight matrix is denoted as *W* = (***w*_1_**, …, ***w*_*L*_**)^⊤^. For patient *i*, denote the maximum observed grade of toxicity type *l* as *G*_*il*_. Then 1(*G*_*il*_ = *h*) is an indicator function which takes value 1 if the maximum observed grade is *h* for toxicity type *l*, and 0 otherwise. The TTP is further normalized to nTTP, $nTTP_{i}=\frac{TTP_{i}}{v}$, in order to constrain the toxicity endpoint within the interval (0, 1), where *v* is a normalization constant.

To construct the TTP score as the toxicity endpoint, it requires input from the physicians involved in the trial to elicit the weight matrix that represents the clinical severity of the selected toxicity. To illustrate, three independent types of toxicities are considered to be related to the treatment and the disease and are therefore solicited: renal, neurological and hematological toxicities. The clinical weight matrix, elicited from the physician, is shown as below. The dose-limiting toxicity (DLT), in this setting, is defined as the occurrence of a grade 3 or 4 renal/neurological toxicity or a grade 4 hematological toxicity.

$$\begin{array}{c} W=(\begin{array}{ccccc} 0 & 0.5 & 0.75 & 1 & 1.5\\ 0 & 0.5 & 0.75 & 1 & 1.5\\ 0 & 0 & 0 & 0.5 & 1 \end{array}). \end{array}$$

Function Tox2nTTP() can be used to compute the nTTP score based on the observed toxicity profile for a patient. For example, for a patient who experiences a grade 1 renal toxicity, grade 2 neurological toxicity and no hematological toxicity, their nTTP score can be computed as follows:

*R> nttp <- Tox2nTTP(tox = c(1, 2, 0),*

*+   wm = matrix(c(0, 0.5, 0.75, 1 , 1.5,*

*+   0, 0.5, 0.75, 1 , 1.5,*

*+   0, 0 , 0 , 0.5, 1 ),*

*+   byrow = T, ncol = 5),*

*+   toxmax = 2.5)*

*R> nttp*

[1] 0.2

The argument tox in the function is used to specify the observed grade for the pre-selected toxicities (range from grade 0 to 4), argument wm gives the elicited weight matrix associated with the selected toxicities; and toxmax specifies the normalization constant. The function returns a value of 0.2 as the nTTP score associated with observed toxicity profile.

### 2.2 Repeated measures design with longitudinal toxicity data

Using a TTP-based toxicity endpoint is an improvement from using the traditional DLT-based endpoint in that it takes into account clinical multidimensionality of multiple types/grades of toxicities for a given toxicity profile, as well as the moderate toxicity events commonly ignored by using DLT endpoint. We further extended it to a repeated measures design (RMD) modeling longitudinal toxicity data with TTP-based endpoints to incorporate toxicity scores from multiple treatment cycles [2].

The **phase1RMD** package uses a linear mixed model. The random intercept is introduced to account for within-patient correlation among repeated toxicity measures. Let *y*_*ij*_ be the nTTP score for patient *i* (*i* = 1, 2, …, *n*) at cycle *j*, where *t*_*ij*_ = *t*_*j*_ = *j* represents the *j*^*th*^ (*j* = 1, 2, …, *J*) cycle of treatment, the same across all patients. We use *x* (*x* = 1, 2, …, *K*) to denote the dose of the agent and *x*_*i*_ is the dose allocated to patient *i*. We assume that the same dose is given to a patient in each cycle of the treatment. The linear mixed effect model with a random intercept for the longitudinal nTTP scores is such that

$$\begin{array}{c} y_{ij}=\beta_{0}+\beta_{1}x_{i}+\beta_{2}t_{j}+\gamma_{i}+\varepsilon_{ij},\;\beta_{1}>0 \end{array}$$ (2)

where ${∊}_{ij}∼N\left(0,\sigma_{∊}^{2}\right)$, $\gamma_{i}∼N\left(0,\sigma_{\gamma }^{2}\right)$, *β*_0_ is the fixed intercept; *β*_1_ represents the dose effect; *β*_2_ represents the cycle effect; *ϵ*_*ij*_ is the measurement error, and *γ*_*i*_ is the random intercept for each patient. Let *θ* represent the parameters to be estimated such that *θ* = (*β*_0_, *β*_1_, *β*_2_, *σ*_*ϵ*_, *σ*_*γ*_)^⊤^.

Patients can have unequal numbers of repeated measures—this is critical as Phase I studies normally suffer from patient dropouts due to DLT or disease progression, and this proposed linear mixed effect model allows us to accommodate differing number of cycles across patients and missing toxicity data beyond cycle 1.

To perform the dose-finding design based on the longitudinal nTTP scores, we start with the lowest dose (assumed as dose level 1 here for illustration), and enroll patients sequentially by cohorts of size *m*. A new cohort of patients will be enrolled when the previous cohort has finished one cycle of treatment. The dose escalation and de-escalation decisions are based on the posterior distributions from fitting the linear mixed model (2) with the accumulated longitudinal toxicity data. Suppose O denotes the current data accumulated; the dose-escalation is guided by Bayesian risk associated with a loss function as below:

$$\begin{array}{c} L(Pi_{Y}\left(x,\theta ,t_{1}\right),Pi_{1})=|Pi_{Y}\left(x,\theta ,t_{1}\right)-Pi_{1}|, \end{array}$$ (3)

where $Pi_{1}$ is the elicited target toxicity in nTTP score in the first cycle of treatment, and $Pi_{Y}\left(x,\theta ,t_{1}\right)$ denote the mean nTTP score given parameter *θ*, at cycle 1 of the treatment for dose level *x*. The dose level *x* that delivers the minimum Bayesian risk, namely, the minimum of the conditional expectation, $E[L(Pi_{Y}\left(x,\theta ,t_{1}\right),Pi_{1})|O]$, will be allocated to the next cohort of patients.

The dose finding algorithm starts with the 3+3 design and switches to proposed model-based design when data on at least two dose levels become available for Bayesian estimation. Skipping un-tested doses is not allowed, and we assume that patients continue the dose assigned on cycle 1 until they exhibit a DLT. The trial will be terminated when a fixed sample size *N* is achieved; and the final recommended MTD is assigned as the dose level minimizing the Bayesian risk based on the complete data.

### 2.3 Repeated measures design with toxicity-efficacy dual endpoints

We further extend the repeated measure design, through jointly modeling the longitudinal toxicity data of nTTP scores and a single early continuous efficacy endpoint [7]. The joint model is comprised of two submodels: a linear mixed effect model for longitudinal nTTP scores as described in (2), and a linear model for a continuous efficacy endpoint, as specified below:

$$\begin{array}{c} E_{i}=\alpha_{0}+\alpha_{1}x_{i}+\alpha_{2}x_{i}^{2}+\nu \gamma_{i}+\epsilon_{e_{i}}, \end{array}$$ (4)

where ${∊}_{e_{i}}∼N\left(0,\sigma_{e}^{2}\right)$, *γ*_*i*_ is the random intercept for the *i*^*th*^ patient, shared across the two submodels, and ${∊}_{e_{i}}$ is the measurement error for efficacy outcome for patient *i*. *θ* represents the parameters to be estimated here: *θ* = (*β*_0_, *β*_1_, *β*_2_, *α*_0_, *α*_1_, *α*_2_, *ν*, *σ*_*ϵ*_, *σ*_*γ*_, *σ*_*e*_)′. The parameter *ν* in (4) measures the strength of association between two submodels. The dose-finding algorithm is three-stage, with the goal to find efficacious doses that are also safe. Suppose *N* is the maximum sample size.

**Stage 1** aims to perform the dose-escalation and define initially allowable (safe) doses in terms of toxicity, based on longitudinal data of nTTP scores only until the sample size of patients enrolled reaches $\frac{N}{2}$. We enroll patients by cohorts of size *m*. The linear mixed effect submodel on toxicity only is used to fit the accumulated longitudinal toxicity data for dose escalation, guided again by Bayesian risk evaluation, where a loss function is defined as in Section 2. The dose level *x* that minimizes the Bayesian risk, will be allocated to the next cohort of patients. Following the notations defined in Section 2, we declare the dose *x* an allowable (safe) dose based on safety if the following two conditions are met:

$$\begin{array}{cc} & P\left\{Pi_{Y}\left(x,\theta ,t_{1}\right)<c_{1}|O\right\}>p_{1}, \end{array}$$ (5)

$$\begin{array}{cc} & P\left\{Pi_{Y}\left(x,\theta ,t_{1+}^{\prime }\right)<c_{2}|O\right\}>p_{2}, \end{array}$$ (6)

where *c*_1_, *c*_2_ are the upper bounds of the mean nTTP score for the first and subsequent cycles of treatment, while *p*_1_, *p*_2_ are the corresponding probability cutoffs respectively. Condition (5) concerns the toxicity at the first cycle of treatment, while condition (6) regards the toxicities for the other late cycles, with $Pi_{Y}\left(x,\theta ,t_{1+}^{\prime }=1\right)$ serving as an aggregate mean nTTP score for treatment cycles beyond cycle 1 at dose level *x*, given the parameter *θ*. These two probability conditions (5) and (6) act here as a stopping rule such that the trial shall be terminated early if there is no allowable (safe) dose. Otherwise, stage 1 continues until the cumulative sample size of patients enrolled reaches $\frac{N}{2}$ and at the end of stage 1, we identify a set of initially allowable doses, denoted by A.

**Stage 2** involves efficacy outcome, and by jointly modeling the toxicity and efficacy submodels, we keep updating the set of allowable doses, A, and randomize patients to the allowable doses with emphasis towards those with higher predicted efficacy for each new cohort. The probability that dose level a∈A is assigned is as follows:

$$\begin{array}{c} \frac{\exp\left\{E[Pi_{E}\left(a,\theta \right)|O]\right\}}{\sum_{x\in A}\exp\{E[Pi_{E}\left(x,\theta \right)|O]\}}, \end{array}$$

where $E[Pi_{E}\left(a,\theta \right)|O]$ is the posterior mean of efficacy outcome, the predicted efficacy, for dose level a∈A. Thus, the higher the predicted efficacy of a dose, the more likely it will be assigned to the next cohort of patients.

**Stage 3** concludes the algorithm when all the data from all treated patients becomes available (i.e., at the end of the trial), where we fit the joint model (2) and (4) to the full data and find the efficacious doses that are also allowable (safe). Let l∈A be the allowable dose that gives the largest posterior mean of efficacy, such that $l=\arg\max_{a\in A}E[Pi_{E}\left(a,\theta \right)|O]$. We define a proximity threshold *δ* such that any dose a∈A that satisfies the condition $|E[Pi_{E}\left(a,\theta \right)|O]-E[Pi_{E}\left(l,\theta \right)|O]|\leq \delta$ will be declared an efficacious dose. We thus define the set of efficacious doses that are also safe, H, such that $H=\{h\in A:|E[Pi_{E}\left(h,\theta \right)|O]-E[Pi_{E}\left(l,\theta \right)|O]|\leq \delta \}$. Any dose h∈H can be picked based on physician’s judgement and preference but in our example in this paper, we choose the lowest (safest) dose in set H as the final recommendation for subsequent phase II or phase III trials for further investigation. Throughout the three-stage design, early termination of the trial is allowed, when there are no allowable (safe) doses in terms of toxicity but skipping of dose levels not previously tried is not allowed at any time.

## 3 Results

### 3.1 Repeated measures design with longitudinal toxicity data

#### 3.1.1 Conducting a trial

In the methods section, we described the statistical framework to conduct a repeated measures design with longitudinal toxicity data. This approach can be implemented using the function RunRMD() in **phase1RMD** package. To install and load the package within R:

*R> install.packages(“phase1RMD”)*

*R> library(phase1RMD)*

The main features of the function RunRMD() will be highlighted using a hypothetical phase I oncology trial. Suppose a drug is to be tested in an oncology phase I trial where the primary objective is to find the MTD. To apply the RMD methods described above, the input from the physicians involved in the trial must be elicited. For example, three types of toxicities are considered related to the drug and will be monitored: renal, neurological and hematological toxicities. Table 1 lists the possible grades for each toxicity type and whether its occurrence is considered a DLT. The physicians are then asked to assign a severity weight for each grade of each toxicity. Given the toxicity types and their severity weights, the target nTTP at cycle 1 must be elicited from the physicians, and is specific to the drug and the cancer type. For illustration purposes, six dose levels have been identified for possible experimentation. The physicians wish to escalate the dose rapidly to the MTD without subjecting many patients to overdose, and wish to enroll patients in cohorts of size three.

**Table 1:** Toxicities and severity weights in the example application.

Toxicity Type	Grade	Severity Weight	DLT
Renal	1	0.5	No
	2	0.75	No
	3	1	Yes
	4	1.5	Yes
Neurological	1	0.5	No
	2	0.75	No
	3	1	Yes
	4	1.5	Yes
Hematological	3	0.5	No
	4	1	Yes

Function RunRMD() is able to interactively conduct a trial with longitudinal toxicity endpoints in nTTP scores from multiple treatment cycles. It implements the linear mixed model to draw the posterior inference on the parameters using Gibbs sampling methods, and use the posterior mean estimates of toxicity at each dose level to make dose recommendation for the next cohort of patients. The recommended dose has the closest toxicity score to the target toxicity score at cycle 1. In order to use function RunRMD(), the user needs to specify the trial parameters, including trlSize: the number of patients enrolled in the trial; sdose: the doses explored in the trial; MaxCycle, the maximum cycles of treatment; and tox.target, the target toxicity at cycle 1 of the treatment.

The user can specify the prior distributions of the model parameters using argument control: beta.dose represents the dose effect, beta.cycle represents the cycle effect, gamma represents the random effect (random intercept), s2.gamma represents the variance of random effects, and s2.epsilon represents the variance of measurement errors. Without historical data to construct priors, non-informative priors can be used so that the estimation is largely dependent on data. The default choice is Normal priors for fixed effects and Inverse Gamma priors for variance parameters, and the users can modify the default priors in the control argument. To update the prior with data and draw posterior inference for the parameters, the MCMC algorithm is implemented, and the user can specify the MCMC parameters including iter: number of MCMC iterations; burnin: number of burn-ins; thin: thinning parameter and chains: number of chains. The parm function specifies the type of the prior distribution and its distribution parameters. Two types of distributions (‘normal’ and ‘invgamma’) are available, for which mean and var parameters are specified for the normal distribution; and shape and scale scale parameters are specified for the inverse gamma (‘invgamma’) distribution.

*R> control <- list(*

*+   beta.dose = parm(“normal”, mean = 0, var = 1000),*

*+   beta.cycle = parm(“normal”, mean = 0, var = 1000 ),*

*+   gamma = parm(“normal”, mean = 0, var = 100 ),*

*+   s2.gamma = parm(“invgamma”, shape = 0.001, scale = 0.001),*

*+   s2.epsilon = parm(“invgamma”, shape = 0.001, scale = 0.001))*

More importantly, function RunRMD() needs the input of toxicity data in order to return the dose for the next cohort of patients in the trial. Data accumulated from a trial can be entered using the data argument, where data represents a data frame specifying patient ID information, dose level, treatment cycle, nTTP score observed from the patient in corresponding treatment cycle, and the DLT event. For example, suppose the following data were collected during the trial:

*R> data(patdata)*

*R> patdata*

*uniqueID  cohort  subj  dose  cycle  nTTP  DLT*

*1 cohort1subj1    1    1    1    1   0.2   0*

*2 cohort1subj2    1    2    1    1   0.0   0*

*3 cohort1subj3    1    3    1    1   0.0   0*

*4 cohort1subj1    1    1    1    2   0.2   0*

*5 cohort1subj2    1    2    1    2   0.0   0*

*6 cohort1subj3    1    3    1    2   0.2   1*

*7 cohort2subj1    2    1    2    1   0.3   1*

*8 cohort2subj2    2    2    2    1   0.2   0*

*9 cohort2subj3    2    3    2    1   0.2   0*

The function RunRMD() prints out the information regarding the trial, gives outputs of posterior estimates (mean, median, standard deviation, and quantiles) of nTTP at each dose each cycle, and returns the recommended dose for the next cohort of patients.

*R> fit <- RunRMD(data = patdata, control = control,*

*+  trlSize = 36, tox.target = 0.28, sdose = 1:6, strDose = 1,*

*+  iter = 2000, burnin = 500, thin = 1, chains = 1)*

Model: RMD with longitudinal toxicity

Doses (skeleton):

1   2   3   4   5   6

The maximum sample size is: 36

The current enrolled number of patients are: 6

The current enrolled cohort is: 2

Posterior estimates (mean) of toxicity:

Dose 1 Dose 2 Dose 3 Dose 4 Dose 5 Dose 6

toxpf1  0.068  0.235  0.402  0.569  0.736  0.904

toxpf2   0.135  0.303  0.470  0.636  0.803  0.970

toxpf3   0.203  0.370  0.537  0.704  0.870  1.038

toxpf4   0.269  0.438  0.604  0.771  0.938  1.105

toxpf5   0.337  0.505  0.670  0.838  1.005  1.172

toxpf6   0.404  0.571  0.738  0.905  1.072  1.240

Next recommended dose: 2

*R> fit $nxtdose*

[1] 2

In the example above, dose 2 is recommended for the next patient cohort as its posterior mean nTTP at cycle 1 is 0.235 closest to the target toxicity of 0.28. To output posterior estimates for a specific cycle, say cycle 2, the user can call the corresponding estimate matrix in the fit object:

*R> fit $tox.est[2,,]*

Dose 1  Dose 2  Dose 3 Dose 4 Dose 5  Dose 6

mean     0.068   0.235   0.402   0.569   0.736  0.904

sd      0.065   0.071   0.141   0.222   0.305  0.390

median   0.069   0.234   0.395   0.557   0.720  0.882

2.5%   -0.057   0.095   0.150   0.180   0.211  0.230

25%     0.026   0.190   0.305   0.415   0.523  0.630

50%     0.069   0.234   0.395   0.557   0.720  0.882

75%     0.112   0.277   0.486   0.709   0.923  1.144

97.5%   0.191   0.386   0.687   1.047   1.396  1.740

**Fig 1:**
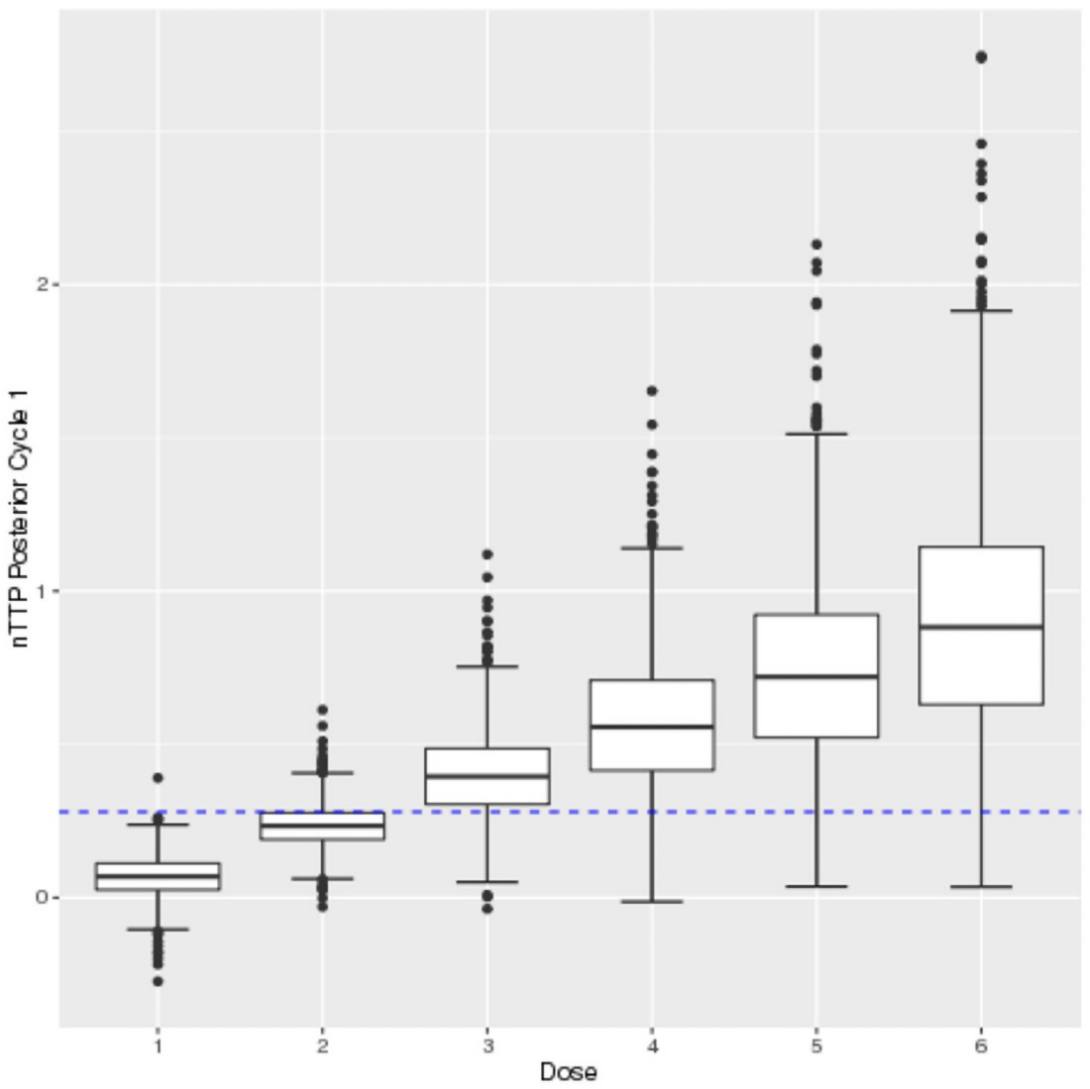
Cycle 1 posterior nTTP.

**Fig 2:**
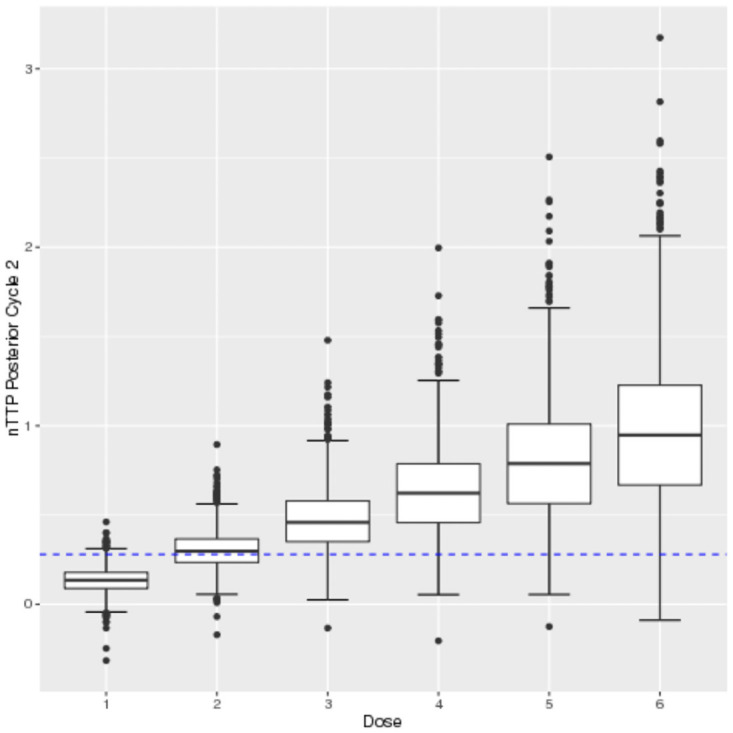
Cycle 2 posterior nTTP.

The argument data is used as input to obtain the next recommended dose as patients are enrolled in the trial sequentially. The function can also be called at the end of the trial to obtain the estimated MTD. To report the results at the end of a trial, plots of the posterior estimates of nTTP are created by using the plot() function. For example plot(fit, cycle = 1) and plot(fit, cycle = 2) displays the results in Figs 1 and 2.

#### 3.1.2 Assessing operating characteristics through simulations

Operating characteristics of clinical trials are often assessed through simulations. In the cases of phase I clinical trials, users are often interested in evaluating the performance of the trial design via the dose allocation percentage and the dose recommendation percentage. Different simulation scenarios of dose-toxicity relationship are often investigated. Phase I data in reality is multi-dimensional; and therefore the process of generating complex but realistic phase I toxicity data is a challenge. In the package, we include two ways to generate independent longitudinal toxicity data using non-parametric and parametric approach, respectively.

For the non-parametric approach, one would need to pre-specify the matrices of probabilities for each type of toxicity for every cycle of the treatment for observing grades 0 to 4 toxicity, for all dose levels. This is possible when we have prior information on the dose-toxicity relationship, for example, from historical data. So the non-parametric approach is used when we have historical data available on the dose-toxicity scenarios under investigation. However, under other circumstances, one would want to run simulations under a large numbers of scenarios, say 100 scenarios. In this case, the parametric approach would be helpful, as we only need to pre-specify the range of the parameters in the proportional odds model.

For the data generation of patient-level toxicity scores, nTTP, under different scenarios, we need to define matrices of probabilities, for each type of toxicity and each cycle of the treatment, of observing grades 0 to 4 toxicity, for all dose levels. Using our previous hypothetical phase I oncology trial example again, assume that six dose levels are investigated of the drug and three types of toxicities are considered to be relevant: renal, neurological and hematological toxicities; and the corresponding clinical severity weights are as given in Table 1. A possible such matrix, where cycle = 1 and toxicity type = renal, looks as below:

$$\begin{array}{c} P_{\text{renal}}=(\begin{array}{ccccc} 0.823 & 0.152 & 0.022 & 0.002 & 0.001\\ 0.791 & 0.172 & 0.032 & 0.004 & 0.001\\ 0.758 & 0.180 & 0.043 & 0.010 & 0.009\\ 0.685 & 0.190 & 0.068 & 0.044 & 0.013\\ 0.662 & 0.200 & 0.078 & 0.046 & 0.014\\ 0.605 & 0.223 & 0.082 & 0.070 & 0.020 \end{array}), \end{array}$$

where grades from 0 to 4 define the columns of the matrix while the dose levels from 1 to 6 define the rows. These probability matrices are used to generate nTTP for each patient, given the dose allocated and the cycle of the treatment. For example, suppose a patient is assigned dose 1 at cycle 1 of the treatment. For the renal toxicity, the first row of matrix *P*_renal_, (0.823, 0.152, 0.022, 0.002, 0.001), corresponds to the probabilities of observing renal toxicity grades 0 to 4 respectively at dose 1. In clinical practice, this is defined by the clinicians based on available literature. In our example, we used the toxicity matrix from the work of [1]. We can thus sample a renal toxicity grade for this patient, according to this probability distribution. We repeat this sampling for the other two types of toxicities and can obtain the toxicity profile (TP) for the patient, for example, grade 1 renal associated with grade 1 neurological and grade 3 hematological toxicities, and can calculate the nTTP score of the patient using function Tox2nTTP(). Such matrices (*P*_renal_) for all three types of toxicities and all treatment cycles define a toxicity structure, where the mnTTP (mean nTTP) for each dose level and cycle can be derived.

The probability matrix is specified through the tox.matrix: tox.matrix is a four-dimension array (Dose x Cycle x Toxicity Type x Toxicity Grade) specifying the probabilities of observing each type of toxicity at each grade (grade ranges from 0 to 4), by each dose level and treatment cycle. An example of tox.matrix is included in the package. If tox.matrix is not provided, the probability matrix can be simulated from a proportional odds model:

$$\begin{array}{c} \text{logit}\left(c_{j}\right)=\lambda_{j}+\zeta *\text{dose}+Psi*\text{cycle},j=0,1,2,3 \end{array}$$ (7)

where the dose effect and cycle effect are specified as *ζ* and Psi respectively. Given a dose, the cycle of the treatment and a particular toxicity type, *c*_*j*_ is the cumulative probability of occurrences of toxicity grades up to *j*. At a given dose and cycle of the treatment, the probability for the occurrence of a certain grade of a certain toxicity type is obtained from taking the corresponding differences between cumulative probabilities. The user can generate tox.matrix by specifying toxtype, intercept.alpha, coef.beta, and cycle.gamma arguments in the GenToxProb() function.

*> tox.matrix <- GenToxProb(toxtype = c(“Renal”, “Neuro”, “Heme”),*

*+   intercept.alpha = c(2, 3, 4.2, 5.7),*

*+   coef.beta = c(-0.2, -0.4, -0.7),*

*+   cycle.gamma = 0)*

Function SimRMD() in the package is able to assess the operating characteristics of the proposed RMD design under different scenarios. It has similar arguments as function RunRMD(), except for a few parameters relating to the simulation. The sample size of the simulated dataset is defined in the trlSize parameter and the size of each patient cohort is defined by chSize. The numTrials parameter specifies the number of simulated trials to assess the operating characteristics. Moreover, the following sets of parameters define the setting of the simulation, in which strDose defines the start dose, sdose defines the vector of available doses and MaxCycle defines the maximum treatment cycle. tox.matrix defines the probability matrix that generate the toxicity data for the corresponding scenario, as demonstrated in Section 3.1.2.

The SimRMD() function prints out the operating characteristics metric such as the percentage of dose allocation and the percentage of dose recommendation. To view the corresponding scenario for the simulation, the user can call the matrix in the fit object that contains the true mean nTTP scores across all cycles at each dose, as well as the true probability of DLT (defined at first cycle) at each dose.

*R> simu <- SimRMD(seed = 2014, strDose = 1, chSize = 3, trlSize = 36,*

*+   numTrials = 100, sdose = 1:6, MaxCycle = 5, tox.target = 0.28,*

*+   control = control, iter = 1000, burnin = 400, thin = 1, chains = 1,*

*+   tox.matrix = tox.matrix)*

Operating characteristics based on 10 simulations:

Sample size 36

$*op*.table

Dose 1 Dose 2 Dose 3 Dose 4 Dose 5 Dose 6

Allocation %   0.091 0.182 0.091 0.273 0.182 0.182

Recommendation % 0.000 0.000 0.110 0.290 0.370 0.230

$sc

Dose 1 Dose 2 Dose 3 Dose 4 Dose 5 Dose 6

mnTTP.1  0.083 0.110 0.146 0.192 0.247 0.309

mnTTP.2  0.083 0.110 0.146 0.192 0.247 0.309

mnTTP.3  0.083 0.110 0.146 0.192 0.247 0.309

mnTTP.4  0.083 0.110 0.146 0.192 0.247 0.309

mnTTP.5  0.083 0.110 0.146 0.192 0.247 0.309

pDLT    0.046 0.066 0.097 0.146 0.221 0.332

### 3.2 Repeated measures design with toxicity-efficacy dual endpoints

#### 3.2.1 Conducting a trial

In addition to the repeated measures design with toxicity endpoint, we also implemented the function RunRMDEFF() to conduct a trial with dual endpoints, incorporating a continuous efficacy outcome and toxicity endpoints in nTTP scores from multiple treatment cycles, according to the approach described above. The function RunRMDEFF() implements MCMC to draw the posterior inference about the parameters using JAGS [5], thus the posterior mean of toxicity and efficacy outcomes are used to estimate their profile at each dose level in the trial. Non-informative priors are used by default so that the estimation is largely dependent on data, but we reserve an option for user to specify a prior. In order to use function RunRMDEFF() the user needs to specify the trial parameters, including trlSize: the maximum number of patients planned for the trial; chSize: the size of each cohort; doses: the doses explored in the trial; MaxCycle: the maximum number of cycles for the treatment; tox.target, the target toxicity at cycle 1 of the treatment. More importantly, the function RunRMDEFF() needs the input of efficacy (efficacy.dat) and toxicity (toxicity.dat) data in order to return the recommended dose for the next cohort of patients as well as the efficacy/toxicity profile for each dose level under investigation in the trial. efficacy.dat, representing the efficacy data, consists of three columns: “subID”, “dose” and “Efficacy” where “subID” consists of a cohort number and a subject number while toxicity.dat specifies the toxicity data, containing subject ID (“subID”), dose (“dose”) allocated to the patient, the treatment cycle (“cycle”), nTTP score (“nTTP”) and lastly DLT event indicator(“DLT”).

The arguments, p1, p2, c1, c2, are used to define allowable (safe) doses through the two probability conditions (5) and (6), representing *p*_1_, *p*_2_, *c*_1_, *c*_2_, respectively. In which, *c*_1_, *c*_2_ are the upper bounds of the mean nTTP score for the first and subsequent cycles of treatment, while *p*_1_; *p*_2_ are the corresponding probability cutoffs respectively. These parameters need to be pre-defined, and should be chosen based on both physicians’ discretion and design’s operating characteristics. Together they are called tuning parameters and are calibrated through simulations. For example, the choices of *c*_1_ and *c*_2_ are based on the trial: if more toxicity is acceptable, then a higher upper bound of toxicity than the target toxicity can be chosen, and *p*_1_ and *p*_2_ can consequently be chosen by simulations to achieve the best operating characteristics. So the probability cutoffs and the toxicity upper bounds would normally go together: increase in the probability cutoff would result in the increase of the toxicity upper boundary. These tuning parameters are common in Bayesian framework when posterior probability/distribution of toxicity is incorporated into the decision rules; for example, Thall and Cook (2004) [8] considered a similar setup in their design with a binary toxicity endpoint. An illustration of such data is given below as well as in the package:

*R> data(eff_dat)*

*R> head(eff_dat)*

subID dose Efficacy

1 cohort1subject1  1 0.014692986

2 cohort1subject2  1 0.005370450

3 cohort1subject3  1 0.004324666

4 cohort2subject1  2 0.005531986

5 cohort2subject2  2 0.300249297

6 cohort2subject3  2 0.002631852

*R> data(tox_dat)*

*R> head(tox_dat)*

subID dose cycle nTTP DLT

1 cohort1subject1  1  1  0.0  0

2 cohort1subject2  1  1  0.0  0

3 cohort1subject3  1  1  0.0  0

4 cohort1subject1  1  2  0.2  0

5 cohort1subject2  1  2  0.0  0

6 cohort1subject3  1  2  0.0  0

The function RunRMDEFF() prints information regarding the trial and outputs a recommended dose for the next cohort of patients, along with the estimated toxicity/efficacy profile for each dose level under investigation, and the set of allowable (safe) doses at the current analysis.

*R> result <- RunRMDEFF(efficacy.dat = eff_dat, toxicity.dat = tox_dat,*

*+   trlSize = 36, doses = 1:6, MaxCycle = 6, tox.target = 0.28,*

*+   p1 = 0.2, p2 = 0.2, c1 = 0.28, c2 = 0.28)*

Model: RMD with longitudinal toxicity

Doses(skeleton):

1    2    3    4    5    6

We are recommending the dose for your next cohort of patients…

The maximum sample size is: 36

The current enrolled number of patients are: 33

The current enrolled cohort is: 11

You are right now in stage 2,

randomizing the next cohort of patients towards higher predicted efficacy…


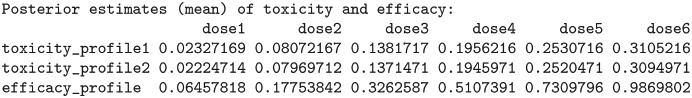


Next recommended dose: 5

*R> result*

$nxtdose

[1] 5


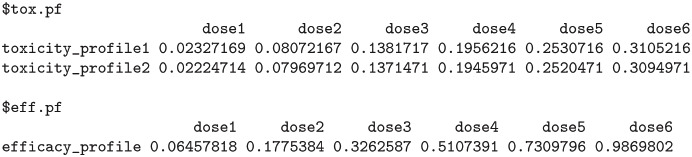


$allow.doses

[1] 1 2 3 4 5

**Fig 3:**
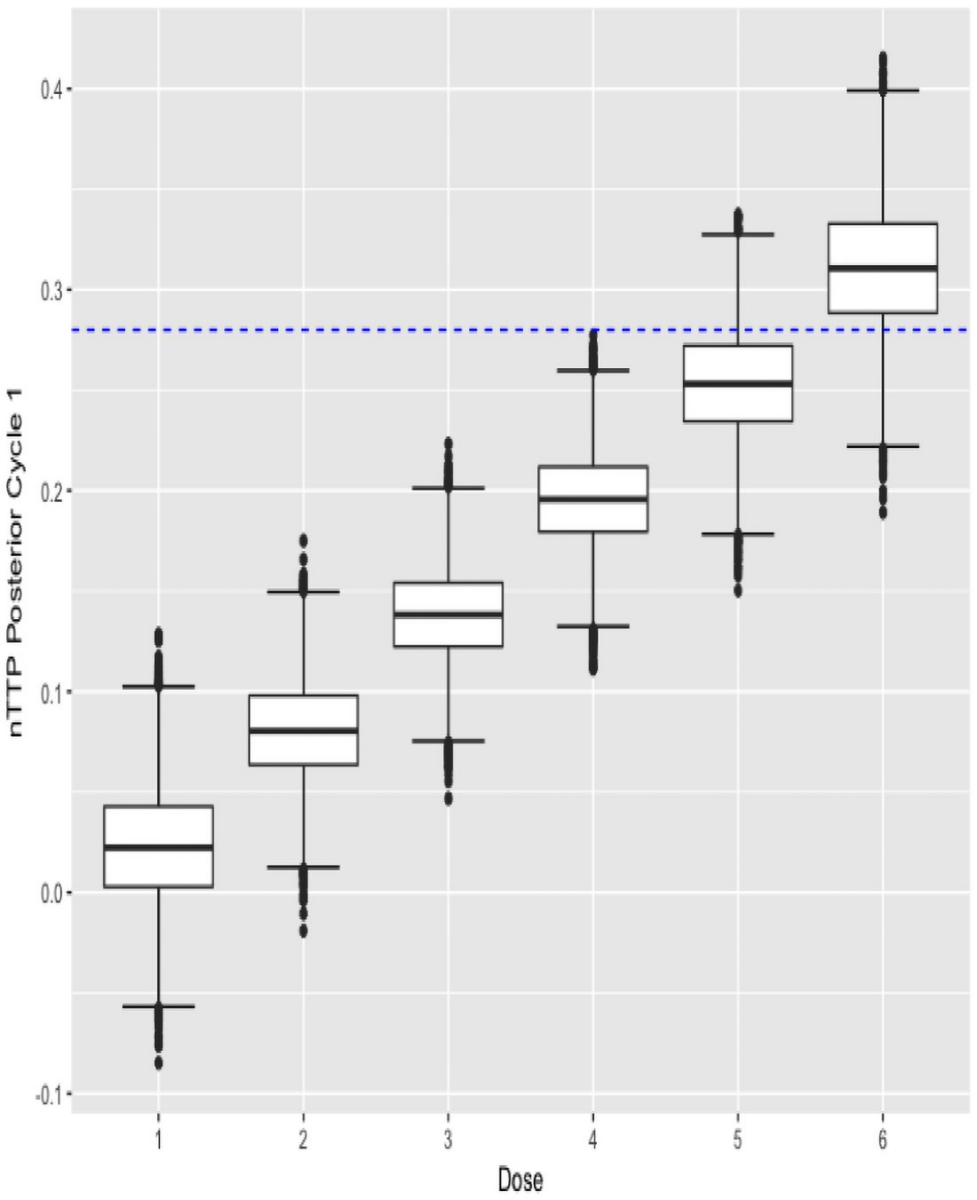
Cycle 1 posterior nTTP.

**Fig 4:**
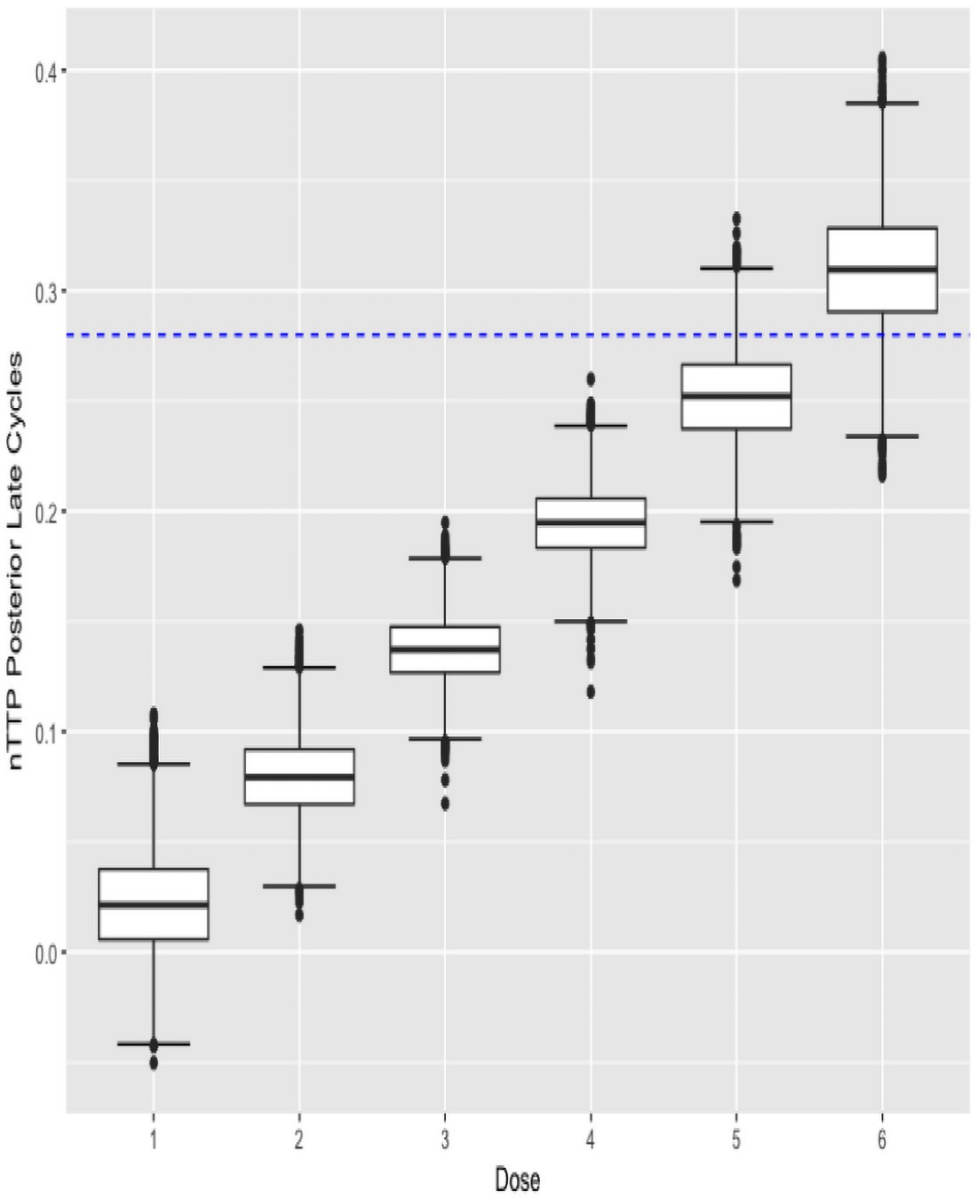
Late cycle posterior nTTP.

**Fig 5:**
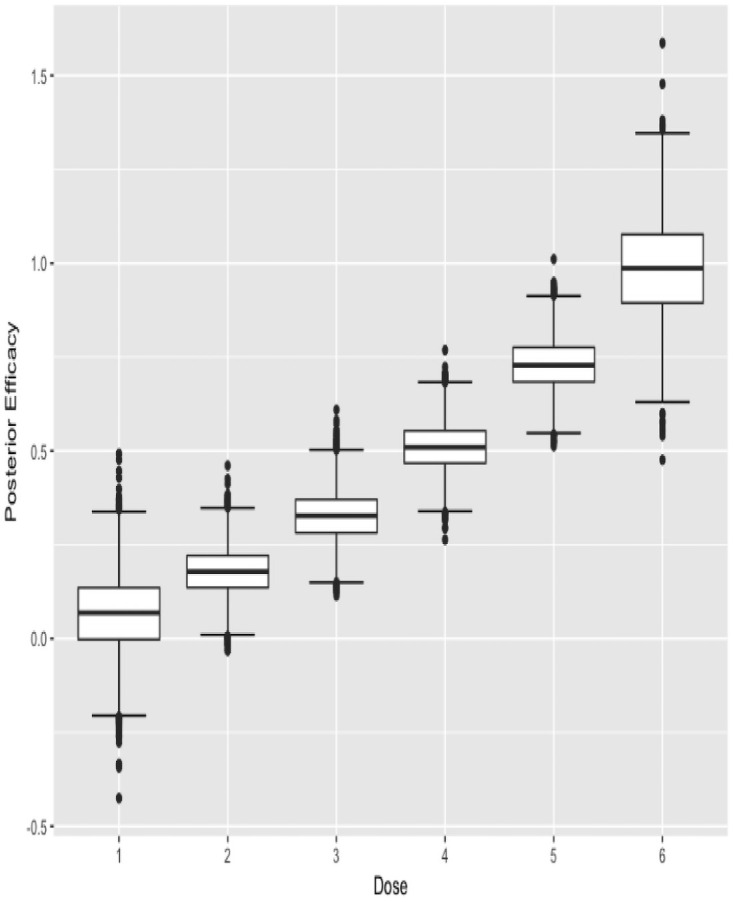
Posterior efficacy.

The function also outputs the boxplots about posterior estimates of nTTP scores for cycle 1 of the treatment as well as for late cycles, and about posterior estimates of efficacy outcome, across each dose under investigation. plot(result$p_nTTP1) and plot(result$p_nTTP2) produce the boxplots about toxicity for cycle 1 of the treatment and late cycles respectively, while plot(result$p_EFF) displays the boxplots about efficacy outcome, as shown below in Figs 3–5.

#### 3.2.2 Assessing operating characteristics through simulations

Through simulation studies, the user can assess how well the design performs under various dose-efficacy-toxicity scenarios by evaluating metrics such as the dose allocation percentage and the dose recommendation percentage.

The generation of longitudinal toxicity data is the same as in Section 3.1.2, where the probability matrices tox.matrix of observing a certain grade of a given toxicity type is either pre-specified or generated via the proportional odds model. Efficacy data, ranging from 0 to 1, is generated from beta distribution given the specified mean efficacy outcome for each dose level and their standard deviations.

Function SimRMDEFF(), in the package, runs simulations for an adaptive, multi-stage phase I dose-finding design incorporating a continuous efficacy outcome and toxicity data from multiple treatment cycles. The function SimRMDEFF() has similar arguments to the function RunRMDEFF() introduced in Section 3.2, except a few parameters relating to the simulation. numTrials specifies the number of simulated trials in the simulation; eff.structure encodes a vector of the mean efficacy outcomes for each dose under investigation, which user can feel free to specify, capturing a wide variety of relationship between efficacy and dose; eff.sd denotes the common standard deviation of efficacy outcome; tox.matrix describes the dose-toxicity relationship, and if it is not provided, it will be simulated via (7). The simulation function also requires the input of wm, the clinical weight matrix for different types and different grades of toxicities, often elicited from physicians, ps1 defining the probability cutoff for defining allowable (safe) doses in stage 1 and toxmax, the normalization constant in computing the nTTP score [1]. The function SimRMDEFF() outputs the scenario information incorporating the mean of efficacy outcome, the mean toxicity profile in nTTP scores across multiple treatment cycles and the probability of DLT events at cycle 1 of the treatment for each dose level under investigation (sc), as well as a table of operating characteristics including dose allocation percentage and dose recommendation percentage (op.table). Below shows an example of the use of function SimRMDEFF().

*R> SimRMDEFF(toxtype = c(“H”, “L”, “M”),*

*+   intercept.alpha = c(1.9, 2.3, 2.6, 3.1),*

*+   coef.beta = c(-0.3, -0.2, -0.25),*

*+   cycle.gamma = 0, tox.target = 0.23,*

*+   thrd1 = 0.23, thrd2 = 0.23,*

*+   eff.structure = rep(0.5, 6),*

*+   p1 = 0.1, p2 = 0.1, ps1 = 0.1,*

*+   numTrials = 100)*

Operating characteristics based on 100 simulations


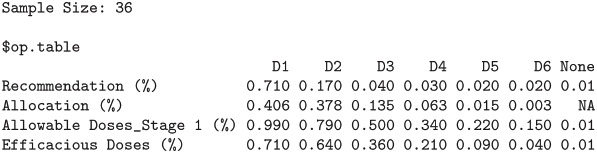


$sc

D1   D2   D3   D4   D5   D6

mnTTP.1st 0.1446 0.1772 0.2151 0.2582 0.3058 0.3572

mnTTP.2nd 0.1446 0.1772 0.2151 0.2582 0.3058 0.3572

mnTTP.3rd 0.1446 0.1772 0.2151 0.2582 0.3058 0.3572

mnTTP.4th 0.1446 0.1772 0.2151 0.2582 0.3058 0.3572

mnTTP.5th 0.1446 0.1772 0.2151 0.2582 0.3058 0.3572

mnTTP.6th 0.1446 0.1772 0.2151 0.2582 0.3058 0.3572

mEFF   0.5000 0.5000 0.5000 0.5000 0.5000 0.5000

pDLT   0.2123 0.2619 0.3201 0.3867 0.4606 0.5395

The example runs 100 simulations, with a flat dose-efficacy pattern where the mean of efficacy does not change with dose, all being equal to 0.5. As is seen from the operating characteristics summarized into op.table, 71% of the simulated trials recommend the lowest dose and another 17% recommend the second lowest dose. More than 80% of the recommendations correspond to the two lowest doses, and these doses are optimal since not only they are the safest (lowest) doses (and lower than the target toxicity), but also they are equally efficacious as the other higher doses.

## 4. Discussion

Phase I clinical trials are designed to identify the recommended phase II dose for future trials. For cytotoxic agents, the recommended dose is generally the maximum-tolerated dose based on dose-limiting toxicity events in the first treatment cycle. Unlike the cytotoxic agents that are often administrated for a limited number of treatment cycles, targeted and immunotherapeutic agents are administered until disease progression and often have a very different toxicity profile, characterized by chronic, prolonged events or cumulative toxicity. Therefore, it becomes important to consider repeated and chronic occurrence of lower grade events, in addition to the early onset of severe adverse events. The idea of using repeated measure of toxicity events have been explored by Legedza and Ibrahim, Braun et al., and Yin et al. [2, 9, 10]. In addition, targeted and immunotherapeutic agents often do not have the monotonically increasing relationship between dose and efficacy. Therefore, incorporating early efficacy signals if available has become important for these novel agents in order to identify the optimal biological dose instead of maximum-tolerated dose. Only limited methods exist for dose-finding designs that account for toxicities and continuous efficacy as dual-endpoints in the selection of recommended phase II dose. Thall and Cook [8] used a set of efficacy toxicity trade-off contours to accommodate outcomes that includes efficacy. Bekele and Shen [11] incorporated the correlation between the binary toxicity and continuous activity outcome via a latent Gaussian variable. Houede et al. [12] used a Gaussian copula to model the marginal outcome and joint distribution of dual toxicity and efficacy endpoints. A common limitation of these designs is that they only considered binary toxicity endpoints, ignoring the fact that toxicity data are high dimensional in nature, with various types, grades, attribution over multiple treatment cycles. To address this, Bekele and Thall (2004) proposed the total toxicity burden (TTB) as the arithmetic sum of different grades and types of toxicity, weighted by the severity weights elicited from clinicians [13]. Lee et al. (2012) proposed the toxicity burden score (TBS) to summarize toxicity using a weighted sum, where the severity weights were estimated via regression using historical data [14]. Ezzalfani et al. (2013) proposed another quasi-continuous toxicity as the Euclidean norm of different grades and types of adverse events, called the total toxicity profile (TTP) [1], upon which our proposed method is based. However, our methods can be easily extended to other types of continuous toxicity score with utilization of the joint model.

In this article, we presented an R package, phase1RMD, to illustrate a novel phase I repeated measures design that we developed to incorporate multivariate patient toxicity profiles from multiple treatment cycles. Furthermore, in studies where preliminary efficacy is evaluated, an adaptive, multi-stage design to identify the most efficacious dose with acceptable toxicity was demonstrated. The repeated measure designs accurately estimated both the magnitude and direction of toxicity trends in late treatment cycles, and allocated more patients at therapeutic doses [2, 15].

To our best knowledge, this is the first software that implement novel phase I dose-finding designs that simultaneously accounts for the multiple-grade toxicity events over multiple treatment cycles and a continuous early efficacy outcome. The R package for implementing these designs is available from the Comprehensive R Archive Network (CRAN). Functions are provided to recommend the next dose based on the data collected in a phase I trial, as well as to assess trial characteristics given design parameters via simulations. With the software published on CRAN, we will pursue the implementation of these designs in phase I trials in real-life settings.

Since the conduct and analysis of clinical trials require rigorous approaches, to facilitate the implementation of our software in the clinical trials, we have taken necessary measures to validate the software and ensure reproducibility. These approaches include:

1. CRAN releases: phase1RMD has been regularly published through CRAN (CRAN 2021) releases https://cran.r-project.org/web/packages/phase1RMD/index.html of updated versions, which enable quality control and testing of the published versions. New features of the software can be implemented and tested on GitHub before these new releases are published to CRAN.
2. Open-source development: The development of phase1RMD is available on a public GitHub.com repository (https://github.com/vivienjyin/phase1RMD). Anybody can freely download the source code, or utilize the other open source development features such as contribution to the codes by opening pull requests.
3. Online documentation and validation report: We utilize the standard documentation generated in CRAN to provide openly accessed manual and validation examples. We also utilize the free GitHub website service to provide validation documentation online. This includes testing example codes and expected testing results in a consistent and easily accessible format.

There are several features yet to be implemented in the package phase1RMD. Future work on the package will focus on allowing intra-patient dose adjustments and flexible definition of decision rules in the application of the clinical trials.

## Supporting information

S1 Text phase1RMD R package validation. (PDF)
