# Supplementary material for: phase1RMD: An R package for repeated measures dose-finding designs with novel toxicity and efficacy endpoints
Source: PLoS One. 2021 Sep 2;16(9):e0256391. doi: 10.1371/journal.pone.0256391 (PMC8412295; doi:10.1371/journal.pone.0256391)
Supplement: S1 Text — (PDF) [file pone.0256391.s001.pdf]

phase1RMD R package validation

Author: Jun (Vivien) Yin

This is the validation report includes testing example data, codes and expected testing results.

Our package is designed to conducted repeated measures design with dual toxicity & efficacy endpoint, which for example can be a continuous efficacy outcome and toxicity endpoints in nTTP scores from multiple treatment cycles. The function `RunRMDEFF` implements MCMC to draw the posterior inference about the parameters using JAGS , thus the posterior mean of toxicity and efficacy outcomes are used to estimate their profile at each dose level in the trial. The following example data is a scenario to validate the function `RunRMDEFF` to recommend next dose. Each subject's dose related efficacy and toxicity are listed as below:

Load an example of the patient efficacy data

```
> #load the patient efficacy data
> data(eff_dat)
> head(eff_dat)
            subID dose    Efficacy
1 cohort1subject1    1 0.014692986
2 cohort1subject2    1 0.005370450
3 cohort1subject3    1 0.004324666
4 cohort2subject1    2 0.005531986
5 cohort2subject2    2 0.300249297
6 cohort2subject3    2 0.002631852
```

Load an example of the patient toxicity data

```
> #load the patient toxicity data
> data(tox_dat)
> head(tox_dat)
            subID dose cycle nTTP DLT
1 cohort1subject1    1     1  0.0   0
2 cohort1subject2    1     1  0.0   0
3 cohort1subject3    1     1  0.0   0
4 cohort1subject1    1     2  0.2   0
5 cohort1subject2    1     2  0.0   0
6 cohort1subject3    1     2  0.0   0
```

Recommend next dose based on toxicity and efficacy data

Using the toxicity and efficacy as in the example above, the function `RunRMDEFF` prints information regarding the trial and outputs a recommended dose for the next cohort of patients, along with the estimated toxicity/efficacy profile for each dose level under investigation, and the set of allowable (safe) doses at the current analysis. The function also outputs the boxplots about posterior estimates of nTTP scores for cycle 1 of the treatment as well as for late cycles, and about posterior estimates of efficacy outcome, across each dose under investigation.

```
> RunRMDEFF(efficacy.dat = eff_dat, toxicity.dat = tox_dat)
Model : RMD with longitudinal toxicity


Doses(skeleton):
    1   2   3   4   5   6


We are recommending the dose for your next cohort of patients...
The maximum sample size is : 36
The current enrolled number of patients are: 33
The current enrolled cohort is: 11
You are right now in stage 2, randomizing the next cohort of patients towards higher predict
Posterior estimates (mean) of toxicity and efficacy:
                        dose1      dose2     dose3     dose4     dose5     dose6
toxicity_profile1 0.02294106 0.08046547 0.1379899 0.1955143 0.2530387 0.3105631
toxicity_profile2 0.02244048 0.07996489 0.1374893 0.1950137 0.2525381 0.3100625
efficacy_profile  0.05793578 0.18123673 0.3351263 0.5196046 0.7346715 0.9803271
Next recommended dose: 5
$nxtdose
[1] 5

$tox.pf
                        dose1      dose2     dose3     dose4     dose5     dose6
toxicity_profile1 0.02294106 0.08046547 0.1379899 0.1955143 0.2530387 0.3105631
toxicity_profile2 0.02244048 0.07996489 0.1374893 0.1950137 0.2525381 0.3100625

$eff.pf
                      dose1     dose2     dose3     dose4     dose5     dose6
efficacy_profile 0.05793578 0.1812367 0.3351263 0.5196046 0.7346715 0.9803271

$allow.doses
[1] 1 2 3 4 5


$eff.pf
```

```
                      dose1     dose2     dose3     dose4     dose5     dose6
efficacy_profile 0.05793578 0.1812367 0.3351263 0.5196046 0.7346715 0.9803271

$allow.doses
[1] 1 2 3 4 5
```

Simulate operating characteristics

Function `SimRMDEFF`, in the package, runs simulations for an adaptive, multistage phase I dose-finding design incorporating a continuous efficacy outcome and toxicity data from multiple treatment cycles. Non-informative priors are used by default so that the estimation is largely dependent on data, but we reserve an option for user to specify a prior. The user can specify the prior distributions of the model parameters using argument `control`: `beta.dose` represents the dose effect, `beta.cycle` represents the cycle effect, `gamma` represents the random effect (random intercept), `s2.gamma` represents the variance of random effects, and `s2.epsilon` represents the variance of measurement errors. Without historical data to construct priors, non-informative priors can be used so that the estimation is largely dependent on data. The default choice is Normal priors for fixed effects and Inverse Gamma priors for variance parameters, and the users can modify the default priors in the `control` argument. To update the prior with data and draw posterior inference for the parameters, the MCMC algorithm is implemented, and the user can specify the MCMC parameters including `iter`: number of MCMC iterations; `burnin`: number of burn-ins; `thin`: thinning parameter and `chains`: number of chains. The `parm` function specifies the type of the prior distribution and its distribution parameters. Two types of distributions: `normal` and `invgamma` are available, for which `mean` and `var` parameters are specified for the normal distribution; and `shape` and `scale`scale parameters are specified for the inverse gamma `invgamma` distribution.

Define prior distribution and toxicity matrix for simulation

```
> #Define prior distributions
> control <- list(
+   beta.dose = parm("normal", mean = 0, var = 1000),
+   beta.other = parm("normal", mean = 0, var = 1000 ),
+   gamma = parm("normal", mean = 0, var = 100 ),
+   s2.gamma = parm("invgamma", shape = 0.001, scale = 0.001),
+   s2.epsilon = parm("invgamma", shape = 0.001, scale = 0.001)
+ )

> #Generate the toxicity matrix
> tox.matrix <- GenToxProb(
+ toxtype = c("Renal", "Neuro", "Heme"),
```

```
+ intercept.alpha = c(2, 3, 4.2, 5.7),
+ coef.beta = c(-0.2, -0.4, -0.7),
+ cycle.gamma = 0)
```

Function `SimRMD` in the package is able to assess the operating characteristics of the proposed RMD design under different scenarios. It has similar arguments as function `RunRMD`, except for a few parameters relating to the simulation. The sample size of the simulated dataset is defined in the `trlSize` parameter and the size of each patient cohort is defined by `chSize`. The `numTrials` parameter specifies the number of simulated trials to assess the operating characteristics. Moreover, the following sets of parameters define the setting of the simulation, in which `strDose` defines the start dose, `sdose` defines the vector of available doses and `MaxCycle` defines the maximum treatment cycle. `tox.matrix` defines the probability matrix that generate the toxicity data for the corresponding scenario.

The `SimRMD` function prints out the operating characteristics metric such as the percentage of dose allocation and the percentage of dose recommendation. To view the corresponding scenario for the simulation, the user can call the matrix in the `fit` object that contains the true mean nTTP scores across all cycles at each dose, as well as the true probability of DLT (defined at first cycle) at each dose.

Simulate trial characteristics, allocation, recommendation rate

```
> #Simulate trial characteristics based on the toxicity matrix
> simu <- SimRMD(seed=2014, strDose=1, chSize=3, trlSize=12,
+ numTrials=1, sdose=1:6, MaxCycle=5, tox.target=0.28,
+ control=control, iter=10, burnin=2, thin=1, chains=1,
+ pathout='./', tox.matrix=tox.matrix)
==================================================================================
Operating characteristics based on 1 simulations:

Sample size 12

$op.table
                 Dose 1 Dose 2 Dose 3 Dose 4 Dose 5 Dose 6
Allocation %      0.333  0.667      0      0      0      0
Recommendation %  0.000  1.000      0      0      0      0

$sc
        Dose 1 Dose 2 Dose 3 Dose 4 Dose 5 Dose 6
mnTTP.1  0.083  0.110  0.146  0.192  0.247  0.309
mnTTP.2  0.083  0.110  0.146  0.192  0.247  0.309
mnTTP.3  0.083  0.110  0.146  0.192  0.247  0.309
```

```
mnTTP.4  0.083  0.110  0.146  0.192  0.247  0.309
mnTTP.5  0.083  0.110  0.146  0.192  0.247  0.309
pDLT     0.046  0.066  0.097  0.146  0.221  0.332
```

phase1RMD implements the following validation measures to facilitate the process of validation and reproducibility:

1. CRAN releases: phase1RMD has been regularly published through CRAN (CRAN 2021) releases https://cran.r-project.org/web/packages/phase1RMD/index.html of updated versions, which enable quality control and testing of the published versions. New features of the software can be implemented and tested on GitHub before these new releases are published to CRAN.

2. Open-source development: The development of phase1RMD is available on a public GitHub.com repository (https://github.com/vivienjyin/phase1RMD). Anybody can freely download the source code, or utilize the other open source development features such as contribution to the codes by opening pull requests.

3. Online documentation & validation report: We utilize the standard documentation generated in CRAN to provide openly accessed manual and validation examples. We also utilize the free GitHub website service to provide validation documentation online. This includes testing example codes and expected testing results in a consistent and easily accessible format.
